# High-resolution transcriptome datasets during embryogenesis of plant-parasitic nematodes

**DOI:** 10.1038/s41597-024-03542-3

**Published:** 2024-06-26

**Authors:** Xueyu Wang, Zhiqing Guo, Dadong Dai, Chuanshuai Xie, Ziwei Zhao, Jinshui Zheng, Ming Sun, Donghai Peng

**Affiliations:** 1 State Key Laboratory of Agricultural Microbiology, Hubei Hongshan Laboratory, Huazhong Agricultural University, Wuhan, 430070 China; 2 College of Life Science and Technology, Huazhong Agricultural University, Wuhan, 430070 China; 3 Hubei Key Laboratory of Agricultural Bioinformatics, College of Informatics, Huazhong Agricultural University, Wuhan, 430070 China

**Keywords:** Sequencing, Embryogenesis

## Abstract

Understanding the transcriptional regulatory characteristics throughout the embryogenesis of plant-parasitic nematodes is crucial for elucidating their developmental processes’ uniqueness. However, a challenge arises due to the lack of suitable technical methods for synchronizing the age of plant-parasitic nematodes embryo, it is difficult to collect detailed transcriptome data at each stage of embryonic development. Here, we recorded the 11 embryonic developmental time-points of endophytic nematode *Meloidogyne incognita* (isolated from Wuhan, China), *Heterodera glycines* (isolated from Wuhan, China), and *Ditylenchus destructor* (isolated from Jinan, China) species, and constructed transcriptome datasets of single embryos of these three species utilizing low-input smart-seq2 technology. The datasets encompassed 11 complete embryonic development stages, including Zygote, 2-cell, 4-cell, 8-cell, 24–44 cell, 64–78 cell, Comma, 1.5-fold, 2-fold, Moving, and L1, each stage generated four to five replicates, resulting in a total of 162 high-resolution transcriptome libraries. This high-resolution cross-species dataset serves as a crucial resource for comprehending the embryonic developmental properties of plant-parasitic nematodes and for identifying functional regulatory genes during embryogenesis.

## Background & Summary

Plant-parasitic nematodes are widely distributed in the ecosystem and have the ability to parasitize various tissues of thousands of plants, causing serious plant diseases and global economic agricultural crop losses^1,2^. The life history of plant-parasitic nematodes is completed within the infected host, where a zygotic cell undergoes a complex series of developmental processes to form a multicellular, functional embryo^3,4^. Crucial events in embryogenesis include reproductive system formation, digestive system determination, stylet formation and neuronal development^5^. These events involve rapid changes and complex regulatory mechanisms in molecular and cellular processes that are essential for the nematode’s parasitism^6^.

Previous research has established distinct variations in early embryonic cleavage patterns across nematode species, highlighting diverse and specific embryonic developmental characteristics that correlate with their phylogenetic positions^7,8^. Notable examples include the plasticity in embryogenesis pattern formation observed in *Acrobeloides nanus*^9^ and the postembryonic mouth dimorphism seen in *Pristionchus pacificus*^10^. Particularly striking peculiarities are observed within the Enoplea group, since members of clade 1B,such as *Enoplus brevis*^11^ and *Pontonema vulgaris*^12^, lack asymmetric cleavages during initial blastomere development, where a gut lineage was evident. Another example is *Tobrilus diversipapillatus*^13^ which displays a unique developmental feature of a prominent coeloblastula, a trait normally thought to be absent in nematodes. This diversity in embryonic development is further reflected in the considerable variability in the time taken by each species to reach equivalent developmental stages. For example, *H. glycines* embryos progress to the J2 stage in approximately six days^14^, while free-living *C. elegans* requires approximately three days to develop from zygote eggs into adults^15^. In contrast, *M. incognita* show a longer development time of up to 20 days^16^. The regulation of embryonic development time is species specific and is regulated by the complex interplay of genes involved in embryonic development. Despite their similar morphology and close genetic relationships, many nematode species exhibit distinct genome and developmental characteristics due to developmental drift or selection^17^. Comprehensive comparative analysis is essential to elucidate the differences between different nematode species.

Comparative transcriptome analysis across species during embryonic development is a valuable approach for elucidating gene expression patterns and understand the evolution of species with interspecific and intraspecific phenotypic variation^18,19^. For example, developmental disparities between *C. elegans* and *C. briggase* were analyzed by comparing the synchronized transition from embryo to adult stage and assessing the mRNA and protein levels changes throughout their life stages^20^. Another study has explored the relationship between embryonic development morphology and gene expression in five free-living nematode species, revealing both similarities and differences in gene expression that underline morphological differences of different species^21^. Moreover, more comprehensive studies spanning multiple nematode species have shed light on the evolutionary aspects of life cycle post-embryonic development. By comparing the developmental transcriptomes of eight nematode species at three developmental stages (embryo, larva and adult), analyzed the evolution of the life cycle after embryonic development^22^. All these studies focused on free-living nematodes species, while the current basic biology research on plant-parasitic nematodes is mainly focused on the parasitic stage, and the embryonic development is more about the description of morphology and development time, lacking data resources on its fine development stage. Thus, analyzing the commonality and specificity of gene expression at different stages of embryonic development across species can provide insight into the evolutionary relationships and courses among different nematode species. Additionally, understanding the embryonic transcriptome information can deepen our knowledge of various biological processes such as cell behavior, signal transduction and gene regulation within organisms, which holds value for understanding the adaptive evolution strategy of nematode defense against parasitic parasites^23^. To achieve this, comprehensive sampling and systematic study of the embryonic development process of different species are required to enhance our understanding of the embryogenesis behavior of plant-parasitic nematodes.

**Fig. 1:**
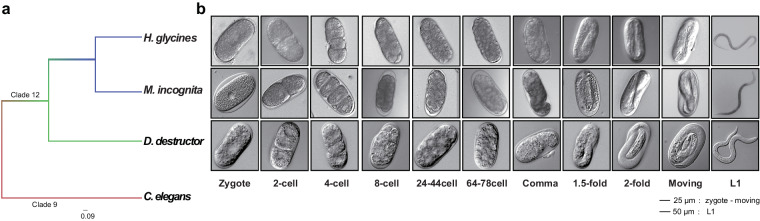
The embryogenesis characteristics of three plant-parasitic nematodes. (**a**) phylogenetic relationships between plant-parasitic nematodes and free-living nematodes species, (**b**) morphological characteristics of embryonic development during zygote to L1 stage.

**Fig. 2:**
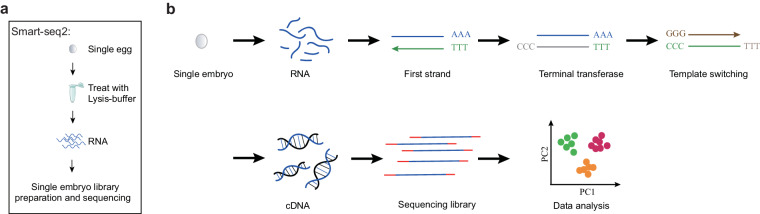
The workflow of single-embryo RNA sequencing based on the smart-seq2 protocol. (**a**) Schematic flow chart of Smart-seq2 library construction, (**b**) the experimental principle of each step.

Here, the migratory endophytic potato tuber nematode (*Ditylenchus destructor*), the sedentary endophytic southern root-knot nematode (*Meloidogyne incognita*), and the sedentary endophytic soybean cyst nematode (*Heterodera glycines*) located on Clade 12 were selected (Fig. 1a). All of them are top-10 plant-parasitic nematodes known for causing significant damage to economic crops^1^. With well-establish laboratory culture, these nematodes can be easily isolation and purification using shallow plant method and sucrose gradient techniques^24,25^. Moreover, their high-quality reference genomes, comprehensive multi-omics functional gene annotations, and extensive basic research make them ideal candidates for dataset utilization and species development analysis. We utilized smart-seq2^26^ (Fig. 2) protocol to conduct single-embryo transcriptomics in three plant-parasitic nematodes. This approach effectively overcame technical challenges related to eggs age synchronization in nematode culture and conducting low-input single egg RNA sequencing. A total of 162 individual embryo libraries across 11 developmental stages were constructed to fill the existing data gap in understanding the fine embryonic developmental stages of plant-parasitic nematodes. These datasets substantially enrich the omics resources available by providing insights into the global gene expression dynamics at each embryonic developmental stage of the three plant-parasitic nematodes, and aim to elucidate the features of cross-species embryonic development.

## Methods

### Experimental design

In the large-scale investigation of nematode populations, the embryonic development of different nematodes was observed to exhibit similar gene expression patterns, primarily dictated by morphological characteristics rather than development time^27,28^. This led to the understanding that different nematode species share commonalities in embryonic development, as indicated by the classical description of embryonic development in nematodes^15^. Consequently, we continuously recorded of the embryonic development phenotypes of *D. destructor*, *M. incognita* and *H. glycines* from zygote stage to L1 larvae, namely Zygote, 2-cell, 4-cell, 8-cell, 24–44 cell, 64–78 cell, Comma, 1.5-fold, 2-fold, Moving, and L1 (Fig. 1b). The information gathered from these developmental stages can be well match with data from other nematode species for further comparative analyses.

### Nematode culture, collection and single embryo isolation

Nematode of the *M. incognita* strain, WHF4-1, were obtained from tobacco roots that had been infected for 35 days. The process of nematode isolation involved, firstly, cleaning and cutting the roots into pieces, followed by the release of the eggs through incubation in 10% sodium hypochlorite for 8 minutes. Subsequently, the mixture was transferred to a 500 μm filter and washed with tap water, followed by washing in a 0.01% Triton X-100 solution. Finally, the purified eggs were collected through centrifugation with 35% sucrose. Similarly, *H. glycines* (strain: Hg5#) nematodes were isolated from infected soybean roots after 35 days. The mature cysts were collected and mechanically disrupted to release the eggs, which were then sterilized with sodium hypochlorite and centrifuged with 35% sucrose to obtain purified eggs for further examination. *D. destructor* (strain: Dd1115) nematodes were isolated from sweet potato tubers after 28 days of infection. The process involved washing the infected potato pieces with sterile water, drying, and cutting them into a shallow dish. A screen of three layers of gauze was used to filter the sweet potato residue, and water was added until the residue was just submerged. The mixture was left at room temperature for 24 hours before collecting and crushing the lumps of sweet potatoes. The purified eggs were then obtained through centrifugation with a 35% sucrose solution.

### Single-embryo isolation and library preparation

Initially, single embryos were transferred into pre-labelled PCR microtubes containing lysis buffer (18 μl 0.3% Triton-X100 + 2 μl RNase inhibitor) measuring 1.5 μl in volume. The low-input mRNA from each of the individual embryos was then subjected to reverse transcription into cDNA using a sensitive highly-multiplexed single-cell RNA-seq (Smart-seq2) protocol. The embryos samples were then carefully cut under a microscope with a scalpel and subjected to rapid centrifugation to initiate the cleavage reaction procedure at 65°C for 10 minutes, followed by an additional incubation at 85°C for 1 minute. Subsequently, the reaction system for the synthesis of the template strand of cDNA was configured, by adding 1 μl of oligo-(dT)30 primer and 1 μl of dNTPs mix to the reaction test tube, followed by incubation at 72°C for 30 seconds to facilitate the specific binding of the PolyA tail. Upon completion of the reaction, the test tube was promptly placed on ice to proceed to the next step. Finally, the synthesis of the first-strand and second-strand reverse transcribed and PCR amplified was carried out in accordance with the Smart-seq2 protocol. The cDNA amplification products were subsequently purified using VAHTS DNA clean beads (Vazyme, CHINA). Following this, 25 μl of beads was added to the reaction system and thoroughly mixed to initiate the purification process, and the mixture was incubated for 8 minutes at room temperature to allow the binding of cDNA to the magnetic beads. With the aid of a magnetic rack, the magnetic beads were separated from the liquid, and the supernatant was carefully removed to clarify the solution. Throughout the process, the PCR tube was kept on the magnetic holder. Subsequently, 200 µl of freshly prepared 80% ethanol was added, and the mixture was incubated for 30 seconds at room temperature. After careful removal, the rinse was repeated. The cleaned-up cDNA was then eluted with 17 µl of Elution Buffer supernatant, resulting in the recovery of a total of 15 µl sample, which was subsequently transferred to a new low-adsorption EP tube and stored at −20 °C.

The library construction process began with a 20 µl reaction, comprising 4 µl of 5 × TTBL, 5 µl of TTE Mix V5, and 5 ng of cDNA (TruePrep DNA Library Prep Kit V2 for Illumina #TD502, Vazyme, CHINA), followed by an incubation at 55°C for 10 minutes. Afterward, 5 µl of 5 × TS solution was added to halt the fragmentation reaction. The addition of index sequences to both ends of the fragmented sequence was carried out using the TruePrep Index Kit V2 for Illumina #TD202, Vazyme, CHINA, with a 10 µl 5 × TAB, 5 µl N5 ×  × , 5 µl N7 ×  × , 1 µl TAE, and a 20 µl reaction volume. Following this, the libraries underwent length sorting (300–700 bp) with DNA clean beads, resulting in a total of 20 mL sample recovery. Subsequently, paired-end sequencing with 150 bp read length was performed using the Illumina NovaSeq 6000 platform.

### Sequencing data saturation test

**Fig. 3:**
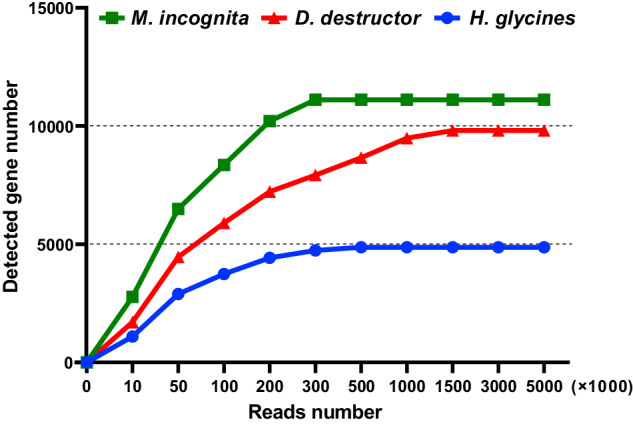
Sequencing data saturation analysis. The X-axis represents the number of randomly selected sampled reads, while the Y-axis shows the number of detected genes.

In order to assess sequencing saturation, samples with the minimum amount of sequencing data volume for each species were selected. The sequencing saturation test involved the examination of samples Dd3_4 (*D. destructor*), Hg1_3 (*H. glycines*) and Mi5_2 (*M. incognita*). Using the seqkit^29^ software (v0.16.1), varying numbers of reads ((10, 50, 100, 200, 300, 500, 1000, 1500, 3000, 5000, (×1000)) were randomly selected for this analysis. The calculation results are shown in Fig. 3. The results indicate that using 3 million reads can achieve saturation and meet satisfy the analysis requirements.

### Gene expression analysis

The reference genome for *M. incognita* WHF4-1 could be found in our previous work^30^, and also could be downloaded at ncbi https://www.ncbi.nlm.nih.gov/biosample/33562119. These datasets are applicable to other publicly reference genome versions as well on the wormbase website. Upon obtaining these genome files, the sequenced reads underwent filtering and trimming using fastp^31^ (v0.20.1). Subsequently, the filtered reads were annotated to their respective species with the specified options (hisat2 -p -x -1 -2 -S –summary-file) using HISAT2^32^ (v2.2.1). Following this preprocessing step, read counts were generated using HTseq^33^. (v0.6.1) and gene expression levels were calculated as transcripts per kilobase million (TPM) using the RSEM^34^ (v1.2.28) command. To enable RSEM to perform these calculations, transcriptome indexes required were prepared using the command rsem-prepare-reference rsem-calculate-expression, with the following options (rsem-calculate-expression –paired-end -no-bam-output –alignments -p). To identify the expressed genes, those with TPM > 1 from at least one time point were selected.

### General statistics and plots

All statistical analyses and plots were conducted in R, using the following packages: ggplot2 (v3.4.0), reshape2^35^ (v.1.4.4), plyr^36^ (v1.8.8), and pheatmap (v1.0.12), unless stated otherwise.

## Data Records

The raw data supporting the study findings for 162 single embryo libraries have been deposited in NCBI with the SRA accession: SRP447238^37^. Additional processed gene count data have been deposited on the GEO: GSE268038^38^. For further inquiry into specific developmental stage data, refer to the accession numbers provided in the Tables 1–3.

**Table 1:** Statistics of the *D. destructor* sequencing data.

Sample	Stages	Total genes detected (TPM ≥ 1)	Overall alignment rate (%)	Accession
Dd1_1	Zygote	7724	56.87%	SRX20871657
Dd1_2	Zygote	7734	43.43%	SRX20871658
Dd1_3	Zygote	7274	44.81%	SRX20871660
Dd1_4	Zygote	8242	41.14%	SRX20871661
Dd2_1	2-cell	8125	42.21%	SRX20871662
Dd2_2	2-cell	7332	42.70%	SRX20871663
Dd2_3	2-cell	9048	48.66%	SRX20871664
Dd2_4	2-cell	8983	55.33%	SRX20871665
Dd2_5	2-cell	8924	58.72%	SRX20871666
Dd3_1	4-cell	8996	55.77%	SRX20871667
Dd3_2	4-cell	10804	60.91%	SRX20871668
Dd3_3	4-cell	10417	60.82%	SRX20871669
Dd3_4	4-cell	10864	63.14%	SRX20871671
Dd3_5	4-cell	9336	70.33%	SRX20871672
Dd4_1	8-cell	8458	61.16%	SRX20871673
Dd4_2	8-cell	9502	65.98%	SRX20871674
Dd4_3	8-cell	9062	65.19%	SRX20871675
Dd4_4	8-cell	8353	67.92%	SRX20871676
Dd4_5	8-cell	9184	65.04%	SRX20871677
Dd5_1	24–44cell	9526	74.57%	SRX20871678
Dd5_2	24–44cell	10774	70.34%	SRX20871679
Dd5_3	24–44cell	10077	74.53%	SRX20871680
Dd5_4	24–44cell	9792	68.84%	SRX20871744
Dd5_5	24–44cell	8514	67.13%	SRX20871745
Dd6_1	64–78cell	10982	74.35%	SRX20871746
Dd6_2	64–78cell	11953	75.29%	SRX20871747
Dd6_3	64–78cell	11927	66.95%	SRX20871748
Dd6_4	64–78cell	10303	70.75%	SRX20871749
Dd6_5	64–78cell	8603	62.79%	SRX20871750
Dd7_1	Comma	9932	72.55%	SRX20871751
Dd7_2	Comma	12162	74.18%	SRX20871752
Dd7_3	Comma	12557	46.47%	SRX20871753
Dd7_4	Comma	9830	49.75%	SRX20871755
Dd7_5	Comma	12703	53.21%	SRX20871756
Dd8_1	1.5-fold	12384	63.55%	SRX20871757
Dd8_2	1.5-fold	11880	68.07%	SRX20871758
Dd8_3	1.5-fold	12201	53.13%	SRX20871759
Dd8_4	1.5-fold	10845	69.61%	SRX20871760
Dd8_5	1.5-fold	9462	74.82%	SRX20871761
Dd9_1	2-fold	12099	57.30%	SRX20871762
Dd9_2	2-fold	11189	71.37%	SRX20871763
Dd9_3	2-fold	12834	66.71%	SRX20871764
Dd9_4	2-fold	15012	74.97%	SRX20871766
Dd9_5	2-fold	8787	69.67%	SRX20871767
Dd10_1	Moving	12959	72.60%	SRX20871768
Dd10_2	Moving	13767	60.23%	SRX20871769
Dd10_3	Moving	13506	55.26%	SRX20871770
Dd10_4	Moving	12242	79.54%	SRX20871771
Dd10_5	Moving	11879	44.07%	SRX20871772
Dd11_1	L1	8615	57.31%	SRX20871773
Dd11_2	L1	10725	55.86%	SRX20871774
Dd11_3	L1	10981	57.49%	SRX20871775
Dd11_4	L1	11883	57.08%	SRX20871683
Dd11_5	L1	9565	59.95%	SRX20871684

**Table 2:** Statistics of the *M. incognita* sequencing data.

Sample	Stages	Total genes detected (TPM ≥ 1)	Overall alignment rate (%)	Accession
Mi1_1	Zygote	15707	91.32%	SRX20871646
Mi1_2	Zygote	19153	91.90%	SRX20871647
Mi1_3	Zygote	16658	63.75%	SRX20871688
Mi1_4	Zygote	16309	91.75%	SRX20871699
Mi1_5	Zygote	18352	89.57%	SRX20871710
Mi2_1	2-cell	25845	93.09%	SRX20871783
Mi2_2	2-cell	21935	94.09%	SRX20871794
Mi2_3	2-cell	22554	48.01%	SRX20871805
Mi2_4	2-cell	16472	40.45%	SRX20871722
Mi2_5	2-cell	20259	81.14%	SRX20871733
Mi3_1	4-cell	17369	90.53%	SRX20871648
Mi3_2	4-cell	17609	91.15%	SRX20871659
Mi3_3	4-cell	16748	45.33%	SRX20871670
Mi3_4	4-cell	13041	41.38%	SRX20871681
Mi3_5	4-cell	20076	86.79%	SRX20871754
Mi4_1	8-cell	20668	47.23%	SRX20871765
Mi4_2	8-cell	23100	87.87%	SRX20871682
Mi4_3	8-cell	20366	78.21%	SRX20871685
Mi4_4	8-cell	20729	86.91%	SRX20871686
Mi4_5	8-cell	20809	92.59%	SRX20871687
Mi5_1	24–44cell	18860	59.16%	SRX20871698
Mi5_2	24–44cell	14862	91.22%	SRX20871690
Mi5_3	24–44cell	16014	91.09%	SRX20871691
Mi5_4	24–44cell	21065	37.65%	SRX20871692
Mi5_5	24–44cell	13958	81.65%	SRX20871693
Mi6_1	64–78cell	14103	82.91%	SRX20871694
Mi6_2	64–78cell	17441	85.28%	SRX20871695
Mi6_3	64–78cell	19373	89.01%	SRX20871696
Mi6_4	64–78cell	32171	56.24%	SRX20871697
Mi7_1	Comma	17729	67.43%	SRX20871698
Mi7_2	Comma	21078	58.98%	SRX20871700
Mi7_3	Comma	19383	74.80%	SRX20871701
Mi7_4	Comma	27580	89.19%	SRX20871702
Mi7_5	Comma	28101	89.56%	SRX20871703
Mi8_1	1.5-fold	23350	93.07%	SRX20871704
Mi8_2	1.5-fold	25229	89.33%	SRX20871705
Mi8_3	1.5-fold	24151	65.22%	SRX20871706
Mi8_4	1.5-fold	8313	77.18%	SRX20871707
Mi8_5	1.5-fold	17332	91.81%	SRX20871708
Mi9_1	2-fold	32042	69.48%	SRX20871709
Mi9_2	2-fold	22314	91.49%	SRX20871711
Mi9_3	2-fold	22300	67.63%	SRX20871712
Mi9_4	2-fold	25344	68.51%	SRX20871713
Mi9_5	2-fold	16179	89.43%	SRX20871776
Mi10_1	Moving	20104	71.30%	SRX20871777
Mi10_2	Moving	21588	91.01%	SRX20871778
Mi10_3	Moving	15438	69.07%	SRX20871779
Mi10_4	Moving	17228	88.22%	SRX20871780
Mi10_5	Moving	22465	93.64%	SRX20871781
Mi11_1	L1	12993	94.83%	SRX20871782
Mi11_2	L1	13868	96.06%	SRX20871784
Mi11_3	L1	15936	95.50%	SRX20871785
Mi11_4	L1	13404	95.51%	SRX20871786
Mi11_5	L1	15443	92.83%	SRX20871787

**Table 3:** Statistics of the *H. glycines* sequencing data.

Sample	Stages	Total genes detected (TPM ≥ 1)	Overall alignment rate (%)	Accession
Hg1_1	Zygote	5500	78.68%	SRX20871788
Hg1_2	Zygote	6299	80.67%	SRX20871789
Hg1_3	Zygote	5391	79.70%	SRX20871790
Hg1_4	Zygote	6635	78.37%	SRX20871791
Hg1_5	Zygote	7470	76.31%	SRX20871792
Hg2_1	2-cell	8037	71.39%	SRX20871793
Hg2_2	2-cell	7507	74.26%	SRX20871795
Hg2_3	2-cell	7789	88.59%	SRX20871796
Hg2_4	2-cell	8639	78.31%	SRX20871797
Hg2_5	2-cell	9378	88.83%	SRX20871798
Hg3_1	4-cell	8021	71.31%	SRX20871799
Hg3_2	4-cell	9820	79.74%	SRX20871800
Hg3_3	4-cell	8866	74.47%	SRX20871801
Hg3_4	4-cell	7383	72.01%	SRX20871802
Hg3_5	4-cell	8685	75.95%	SRX20871803
Hg4_1	8-cell	8548	63.19%	SRX20871804
Hg4_2	8-cell	8033	91.17%	SRX20871806
Hg4_3	8-cell	9096	65.62%	SRX20871807
Hg4_4	8-cell	8355	91.09%	SRX20871714
Hg4_5	8-cell	8560	81.49%	SRX20871715
Hg5_1	24–44cell	9431	73.69%	SRX20871716
Hg5_2	24–44cell	8288	80.42%	SRX20871717
Hg5_3	24–44cell	9275	62.95%	SRX20871718
Hg5_4	24–44cell	9450	80.22%	SRX20871719
Hg5_5	24–44cell	9534	79.18%	SRX20871720
Hg6_1	64–78cell	9601	89.93%	SRX20871721
Hg6_2	64–78cell	10369	92.19%	SRX20871723
Hg6_3	64–78cell	9078	79.22%	SRX20871724
Hg6_4	64–78cell	10576	77.29%	SRX20871725
Hg6_5	64–78cell	9112	79.49%	SRX20871726
Hg7_1	Comma	9802	67.68%	SRX2087127
Hg7_2	Comma	11625	70.89%	SRX20871728
Hg7_3	Comma	8362	71.84%	SRX20871729
Hg7_4	Comma	13551	76.27%	SRX20871730
Hg7_5	Comma	8370	73.19%	SRX20871731
Hg8_1	1.5-fold	11313	79.81%	SRX20871732
Hg8_2	1.5-fold	12079	69.06%	SRX20871734
Hg8_3	1.5-fold	12811	67.53%	SRX20871735
Hg8_4	1.5-fold	8066	81.24%	SRX20871736
Hg8_5	1.5-fold	11230	78.02%	SRX20871737
Hg9_1	2-fold	12022	81.21%	SRX20871738
Hg9_2	2-fold	9193	80.95%	SRX20871739
Hg9_3	2-fold	12686	81.13%	SRX20871740
Hg9_4	2-fold	10909	82.22%	SRX20871741
Hg10_1	Moving	13001	83.53%	SRX20871742
Hg10_2	Moving	10812	76.39%	SRX20871743
Hg10_3	Moving	10481	78.70%	SRX20871649
Hg10_4	Moving	8010	86.89%	SRX20871650
Hg10_5	Moving	8803	78.74%	SRX20871651
Hg11_1	L1	10324	89.72%	SRX20871652
Hg11_2	L1	8889	78.25%	SRX20871653
Hg11_3	L1	9616	88.77%	SRX20871654
Hg11_4	L1	9608	86.28%	SRX20871655
Hg11_5	L1	9482	89.64%	SRX20871656

## Technical Validation

**Fig. 4:**
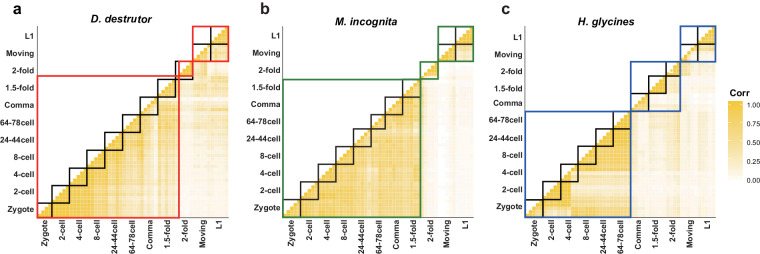
Test of correlation of data between each sample replicate about three species. The analysis included filtered gene sets only if their TPM ≥ 1. The quality of the data was assessed using replicates from the same stage for biological replicate comparisons, revealing a high correlation in expression values between biological replicates of each species. (**a**–**c**) plant-parasitic nematodes generally showed a high correlation between the early and middle stages of embryonic development, samples from the zygote, 2-cell, 4-cell, 8-cell and 24–44 cell stages clustered closely, indicating that early developmental transcriptional profiles are similar.

**Fig. 5:**
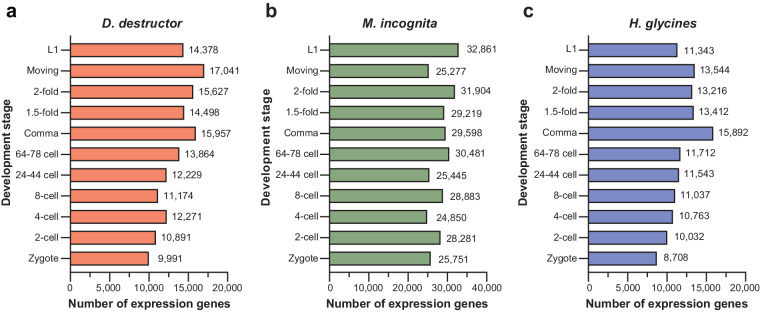
Statistical analysis of gene expression in three plant parasitic nematodes at different stages of embryo development. The X-axis represents the embryonic development stages, while the Y-axis shows the number of expressed genes. Genes that had an average TPM ≥ 1 across all biological replicates for at least one stage in each developmental phase were considered expressed genes, enabling an examination of overall gene expression in each stage.

The transcriptome datasets outlined in this study were optimized using established single nematode sequencing technology. In the preliminary experiment, we encountered challenges in achieving a clean cutting operation on small individual nematode eggs, attributed to their smooth egg shells. Consequently, we conducted repeated sampling of 10–20 individual samples in each period and subsequently filtered down to 4 to 5 replicates for further analysis. All input cDNA concentrations were rigorously determined using Qubit^®^ 3.0 Fluorometer, followed by library construction with a cDNA starting amount of 5 ng using Tn5 transposase. Subsequent to quality control, involving the removeal of adapters, it was found that 50% to 90% of the reads in each library could be successfully mapped to the reference genome (Tables 1–3). To assess the repeatability of the data, we computed the TPM value of each gene in every sample and performed clustering through correlation coefficient, the analysis results showed a high correlation between duplicate samples and adjacent development stages, especially evident in the cell stages of the three species (Fig. 4). Notably, we observed significant fluctuations in different samples during the gastrula development period, indicating that the embryo development underwent pronounced fluctuations, indicative of a transitional process between distinct developmental stages, as depicted in Fig. 4. In order to assess the validity of the data, the number of genes expressed at each developmental stage in different species was quantified, offering a preliminary insight into gene dynamic expression as a valuable data resource (Fig. 5). The processed gene count data file has been uploaded to the GEO database, and the resulting information has been tabulated (Tables 1–3). After quality control and alignment verification, the datasets successfully achieved the capture of gene expression dynamics, providing a feasible basis for later multi-version genome alignment.

## Data Availability

No custom software code was developed for this study. All bioinformatics tools and pipelines are Follow the manuals and protocols provided by the respective software developers. The software and version have been thoroughly described in the Methods section.
